# Highly flexible, wearable, and disposable cardiac biosensors for remote and ambulatory monitoring

**DOI:** 10.1038/s41746-017-0009-x

**Published:** 2018-01-25

**Authors:** Stephen P. Lee, Grace Ha, Don E. Wright, Yinji Ma, Ellora Sen-Gupta, Natalie R. Haubrich, Paul C. Branche, Weihua Li, Gilbert L. Huppert, Matthew Johnson, Hakan B. Mutlu, Kan Li, Nirav Sheth, John A. Wright, Yonggang Huang, Moussa Mansour, John A. Rogers, Roozbeh Ghaffari

**Affiliations:** 1 MC10 Inc, Lexington, MA 02421 USA; 2 Massachusetts General Hospital, Boston, MA 02114 USA; 3 AML, Department of Engineering Mechanics, Center for Mechanics and Materials, Tsinghua University, Beijing, 100084 China; 4 Department of Civil and Environmental Engineering, Mechanical Engineering, and Materials Science and Engineering, Northwestern University, Evanston, IL 60208 USA; 5 Center for Bio-Integrated Electronics, Departments of Materials Science and Engineering, Biomedical Engineering, Chemistry, Mechanical Engineering, Electrical Engineering and Computer Science, and Neurological Surgery, Simpson Querrey Institute for Nano/Biotechnology, McCormick School of Engineering, Northwestern University, Evanston, IL 60208 USA

**Keywords:** Electronic devices, Translational research

## Abstract

Contemporary cardiac and heart rate monitoring devices capture physiological signals using optical and electrode-based sensors. However, these devices generally lack the form factor and mechanical flexibility necessary for use in ambulatory and home environments. Here, we report an ultrathin (~1 mm average thickness) and highly flexible wearable cardiac sensor (WiSP) designed to be minimal in cost (disposable), light weight (1.2 g), water resistant, and capable of wireless energy harvesting. Theoretical analyses of system-level bending mechanics show the advantages of WiSP’s flexible electronics, soft encapsulation layers and bioadhesives, enabling intimate skin coupling. A clinical feasibility study conducted in atrial fibrillation patients demonstrates that the WiSP device effectively measures cardiac signals matching the Holter monitor, and is more comfortable. WiSP’s physical attributes and performance results demonstrate its utility for monitoring cardiac signals during daily activity, exertion and sleep, with implications for home-based care.

## Wearable electronics: digital fingers on the pulse

A highly flexible, low-power wearable sensor that harvests energy and monitors cardiac signals has been developed by Lee et al. The team was led by Dr. Roozbeh Ghaffari and co-workers at MC10 Inc. and Northwestern University’s Center for Bio-Integrated Electronics at the Simpson & Querrey Institute, in collaboration with the Massachusetts General Hospital and Tsinghua University. The novel wearable sensors measure cardiac signals comparable in signal fidelity to those achievable with expensive monitoring systems used in hospitals. Wearable health-care solutions are fundamentally changing the way we monitor our well-being at all times of the day, no matter whether we are asleep at home or busy at work. The sensors reported here are lightweight, inexpensive to manufacture, robust to everyday use, and capable of wireless data transmission and energy harvesting to and from a smartphone. The approach proved successful for measuring episodic electrocardiograms (ECG) and continuous heart rate signals with significantly higher patient comfort scores compared to standard Holter monitors in an initial pilot study conducted at the Massachusetts General Hospital (MGH).

## Introduction

Wearable biosensing systems have become ubiquitous, providing effective routes for quantifying important physiological metrics in both medical and consumer applications.^1–5^ Multi-lead Holter monitoring devices and event monitors, for example, represent the clinical standard of care for detecting and diagnosing cardiac rhythm^6^ and rate disorders based on continuous electrocardiogram (ECG) waveforms and rate-related information.^7–10^ Although these devices have been widely adopted, they are susceptible to poor patient compliance due in part to their bulky form factor and wired connections to leads.^11,12^

Advances in electronics miniaturization and semiconductor performance have significantly reduced the areal footprint and overall size of wearable sensors, creating new market opportunities for consumer and medical health monitoring.^13^ Single-lead cardiac devices^14,15^ have enabled continuous ECG monitoring with significantly smaller footprints compared to Holter monitors, but these patch-based devices contain packaged electronic components and are mechanically rigid,^12,16^ thus limiting intimate skin coupling for repeated, long-term daily use. Increasing interest in quantifying cardiac metrics at home has led to the integration of low profile and high performance heart rate sensors in apparel and wrist bands, but these devices usually rely on photoplethysmography (PPG) and tend to be limited in signal accuracy compared to the clinical standards of care.^17^ Poor accuracy is caused by the physical limitations of PPG, magnified by inherent challenges associated with the peripheral wrist location of the sensor and noisy interface with the skin.^18^ Despite these limitations, the convenience of wrist-worn devices have led researchers to estimate ECG parameters from PPG signals,^19^ demonstrating that there is a continued desire to measure cardiac rhythm metrics with improved patient comfort.

Recent advances in flexible epidermal electronics technologies have begun to address many of the mechanical, wearability and comfort limitations attributed to existing classes of wearable and apparel-based cardiac and heart rate monitoring devices.^20–34^ These novel epidermal devices contain soft, conformal sensors and associated circuits embedded in ultrathin encapsulating layers that achieve intimate skin coupling.^25,34^ Here, we present a highly flexible epidermal design and clinical implementation of a novel ECG and heart rate logging wearable sensor, henceforth referred to as “WiSP”, which is low cost, light-weight (1.2 g), and capable of energy harvesting. The WiSP device is comparable in size to a standard adhesive bandage (58 mm × 25 mm × 1 mm) and streams physiological data to commercial smartphones via standard near-field-communication (NFC) for use in both ambulatory and home-based settings.

## Results

### WiSP device design and characterization

**Fig. 1:**
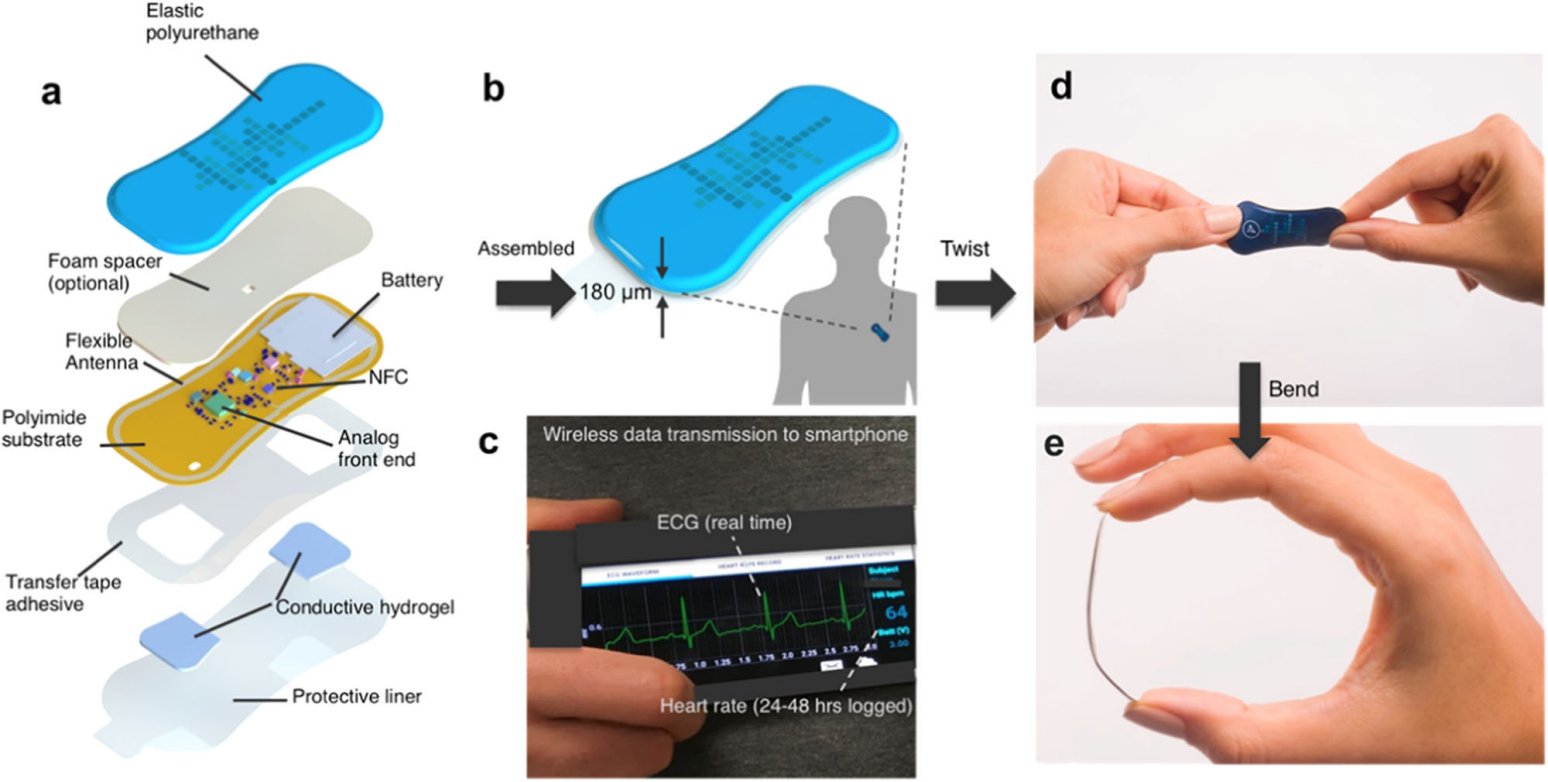
Schematic illustrations and images of soft flexible cardiac sensor in a thin elastic enclosure and data transfer to smartphone app. **a** Exploded image of WiSP device showing the multiple polymeric, electronic, adhesive and hydrogel layers. Image was created by co-author Don E. Wright (permission granted). **b** Illustration of assembled WiSP device consisting of five distinct layers (180 µm thick along the edges, 1.2 g) attached to the torso (in lead I or lead II orientation). The human silhouette is based on an icon created by Freepik (permission granted). **c** Data are wirelessly transmitted to smartphone (via NFC) for visualization of logged heart rate data and/or real-time ECG waveforms, and subsequently transmitted from smartphone to a cloud server (via WiFi or cellular connectivity). **d**, **e** WiSP device in mechanical twist and bend deformations

The WiSP epidermal sensor design in Fig. 1a, b highlights the key structures, as well as the multi-layered conformal mechanics that lead to tight skin coupling and robust cardiac signals. An elastic layer of polyurethane serves as an encapsulating layer. Measurements of water ingress rate under full immersion conditions show no water permeability over several hours, with no changes in WiSP device performance. The electronics are electrically and mechanically coupled to a flexible substrate layer that includes the electroless nickel immersion gold electrodes. The user-facing side of the device is covered with a biocompatible, medical grade skin adhesive and selectively coated conductive hydrogels for direct skin contact. A significant fraction of the skin-side surface area is dedicated to the two electrodes. They are spaced apart sufficiently^35^ to allow for capture of the full PQRST waveforms (Fig. 1c), yet mechanically flexible (Fig. 1d, e).

**Fig. 2:**
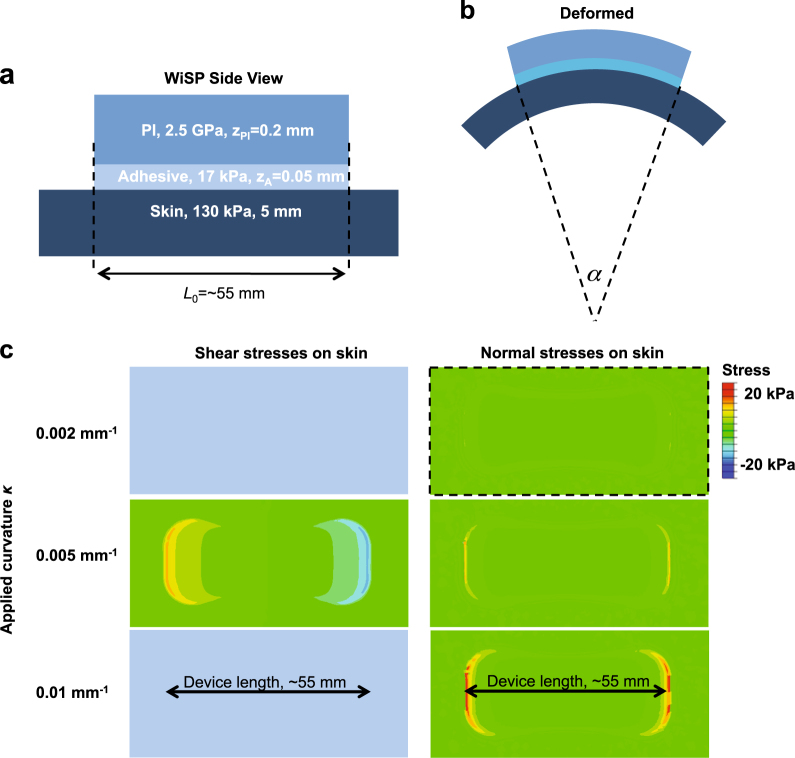
Summary of computational studies detailing the effects of normal and shear mechanical stresses from WiSP device on human skin. **a** Schematic drawing illustrating the cross-sectional design of simplified WiSP device attached to human skin with key lateral and transverse dimensions (*z*_tot_ = 0.25 mm and *L*_*o*_ = 55 mm) and material layers (adhesive layer and PI layer) and their associated Young’s modulus, *E*. **b** Side profile view of WiSP device attached to skin surface under bending deformation with angle *α*. The applied curvature (*κ* = *α*/*L*_o_) is defined by the curvature of the skin under the device. **c** Finite element (FE) simulation results for a device applying shear (left column) and normal (right column) stresses on skin during bending deformations. For 0.002 and 0.005 mm^−1^ applied curvatures, the interfacial stresses on the skin are ~20 kPa, which is within the range of normal skin sensitivity. For 0.01 mm^−1^ applied curvatures, the interfacial stresses on the skin exceed 20 kPa near perimeter of the WiSP device

We applied ABAQUS commercial software^36^ to study the bending response of a simplified WiSP device during wear on the skin (Fig. 2a, b). The simplified WiSP device, consisting of a polyimide (PI) layer with elastic modulus ~2.5 GPa and Poisson’s ratio of 0.34, was mounted on a skin substrate (elastic modulus ~130 kPa and Poisson’s ratio 0.5; ref. ^37^) through a 0.05 mm-thick adhesive layer (elastic modulus ~17 kPa and Poisson’s ratio 0.5; ref. ^38^). Supplementary Fig. S1 shows that the interfacial stresses (particularly normal stresses) on the skin increase as the PI thickness increases under a fixed applied curvature of 0.005 mm^−1^ (defined by the curvature of the skin under the device, *κ* =* α*/*L*, as shown in Fig. 2a, b). These results indicate that thin form factors (e.g., ~0.2 mm PI thickness) for WiSP are well matched to the mechanical properties and curvature of the body.

With an average PI thickness of 0.2 mm for WiSP in the model, Fig. 2c shows the interfacial stresses between the adhesive layer and skin under different applied curvatures (0.002, 0.005, and 0.01 mm^−1^). The interfacial stresses on skin are largest near the perimeter of the WiSP device, which are within the threshold for normal skin perception^34^ (20 kPa) for applied curvatures of 0.002 and 0.005 mm^−1^. In response to aggressive applied curvatures (~0.01 mm^−1^), the interfacial stresses on skin only exceed 20 kPa at a small local area (Fig. 2c), and the maximum principal strains on the top surface of the PI layer (where the electronics are located) are smaller than 0.2% (Supplementary Fig. S2). As a result, the flexible design of WiSP remains functional and comfortable during bending, and unlikely to experience damage in the active electronics layers during wear.

To ensure reliable heart rate computations and ECG capture, the WiSP onboard hardware conditions biopotentials with an instrumentation amplifier configured to amplify time varying signals and attenuate common-mode signals. A second-order high-pass filter and an amplified second-order low-pass active filter then condition the signal. A right-leg drive circuit servos the common-mode voltage at the electrodes and improves common-mode rejection. Ancillary active and passive components provide leads-off detection. To further condition cardiac signals, the microcontroller with integrated ADC oversamples the signal and feeds it into a 25-tap IIR digital filter. A state-machine computes heart rate, logs data, streams live ECG, controls LEDs and manages NFC, data encryption and writing to memory. A learning algorithm measures the ECG baseline level, identifies aspects of the ECG morphology, detects R-wave signatures, and computes heart rate based on the subject’s personal ECG signature.

The WiSP microcontroller also optimizes power consumption by coordinating power harvesting from NFC, system activation, wake, sleep, and power down. Empirically tested switching times control the surge current from the load at system activation and ensure reliable startup of the WiSP device from either the battery or NFC power harvester. Once the system is initialized, active components that are not being used at any given time enter a low-power state and are initiated again only when needed. To remove the need for bulky physical buttons, the WiSP device relies on wireless activation using an NFC-enabled smartphone. By positioning the smartphone in close proximity to the WiSP device (~3 cm), the NFC field is detected by an NFC integrated circuit and a dedicated circuit that activates the system. The microcontroller then confirms whether activation is valid, and, in turn, continues to power-up. A similar set of actions can wirelessly shut down the WiSP.

A custom-designed primary cell battery (450 µm thick; 4 mAh capacity) supplies power to the electronics and memory module for logging heart rate. The logged data may be retrieved with or without battery power. Likewise, the full ECG waveform may be streamed to the smartphone solely using harvested NFC power. The system uses end-to-end encryption from the patch to the smartphone, and from the smartphone to a cloud server. This NFC functionality is interoperable with most smartphones (ISO/IEC 14443 Part 2 and 3 compliant) and takes advantage of the custom-designed flexible antenna that wraps around the circumference of the WiSP device. The antenna and system design ensure reliable operation despite variation among different smartphones’ NFC broadcast power. The flexible antenna was designed to operate efficiently on both soft tissue and in free air to ensure usability and reliability across use cases. Tests verify that WiSP works with a wide variety of commercially available smartphones, allowing consumers and/or patients to use WiSP with their own smartphones at the home (Supplementary Table S1).

### Heart rate and ECG measurements

**Fig. 3:**
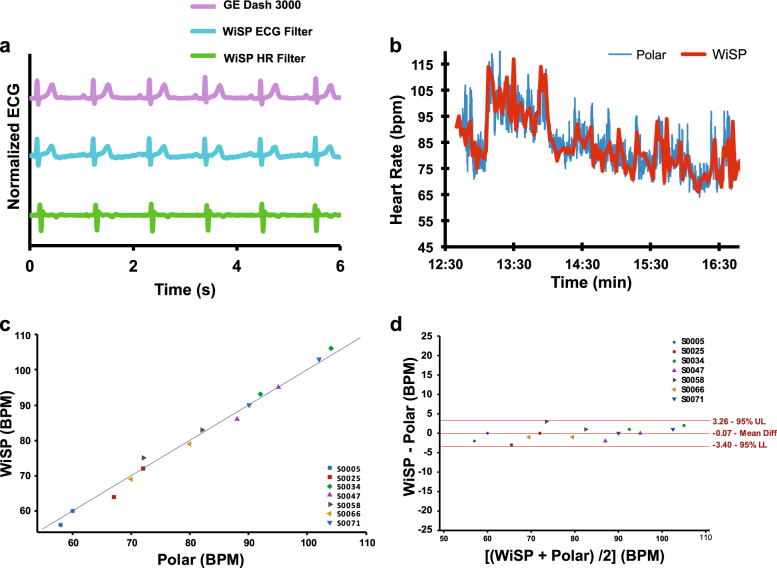
Cardiac sensing data with WiSP compared to control devices in healthy subjects. **a** Plot of ECG waveforms simultaneously recorded from GE Dash 3000 and WiSP with ECG and heart rate (HR) management filter settings. **b** Plot of heart rate data simultaneously recorded with Polar H7 and WiSP devices on a healthy subject during daily activity. **c** Scatter plot of measured heart rate data (*n* = 7 subjects) for WiSP and Polar H7 devices. **d** Bland–Altman analysis of WiSP vs. Polar H7 (*n* = 7 subjects) showing data sets falling within +3.26 BPM (upper limit: UL) and −3.40 BPM (lower limit: LL)

The WiSP ECG waveforms were captured from healthy subjects and compared to measurements taken concurrently with a clinical standard of care product (Fig. 3a; GE Dash 3000 Patient Monitor). The WiSP device is capable of capturing full, differential ECG signals across lead I, II, or III anatomical positions. Figure 3a shows representative WiSP ECG waveforms using two distinct filter settings tuned for ECG and heart rate signal capture, respectively. The ECG filter allows for visualization of P and T-waves (middle trace of Fig. 3a), whereas the heart rate filter (bottom trace of Fig. 3a) operates with a narrow pass-band, thereby eliminating P and T-wave morphology as well as most motion and muscle activation artifacts. By applying this heart rate filter, we could test R-wave detection algorithms and benchmark against existing heart rate sensors.

A field study (Fig. 3b) conducted on healthy subjects provides base-level validation for the WiSP hardware, adhesive, and wear locations. In this validation test, we attached the WiSP and a commercially available Polar H7 heart rate monitor to the chest of each healthy volunteer (>4 h) and recorded heart rate data during routine daily activities. The Polar H7 is a chest-strap electrode-based, heart rate monitor with accuracy higher than wrist-worn monitors and comparable to that of ECG systems.^17^ Heart rate data was collected from both devices at 2-min intervals over this time duration as the basis of the comparison (Fig. 3c, d). The WiSP and Polar data strongly correlated with 95.4% of the two data sets falling within +3.26 BPM and −3.4 BPM (Fig. 3d) across *n* = 7 healthy subjects.

### Remote monitoring study of patients with atrial fibrillation (AF)

To test the signal fidelity, usability, and wearability of the WiSP device, we conducted a clinical study for remotely monitoring heart rate parameters in a cohort of patients with AF. In total, 17 AF patients were instrumented with a WiSP device and Holter monitor (Philips Inc., DigiTrak XT) and released from the clinical setting. The average age of the patients was 66.9 years; 88% were male, 12% were female, and their average BMI was 31.5 (Table 1).

**Table 1:** Patient demographics for 24-h clinical study comparing WiSP device and Holter monitor

Variables		Subjects (*n *= 17)	
Age (years)	Average	66.88	
	Std. Dev	9.72	
	Min	41	
	Max	82	
Gender	Male	15	88%
	Female	2	12%
BMI	Average	31.5	
	Std. Dev	6.22	
	Min	21.47	
	Max	42.04	

**Fig. 4:**
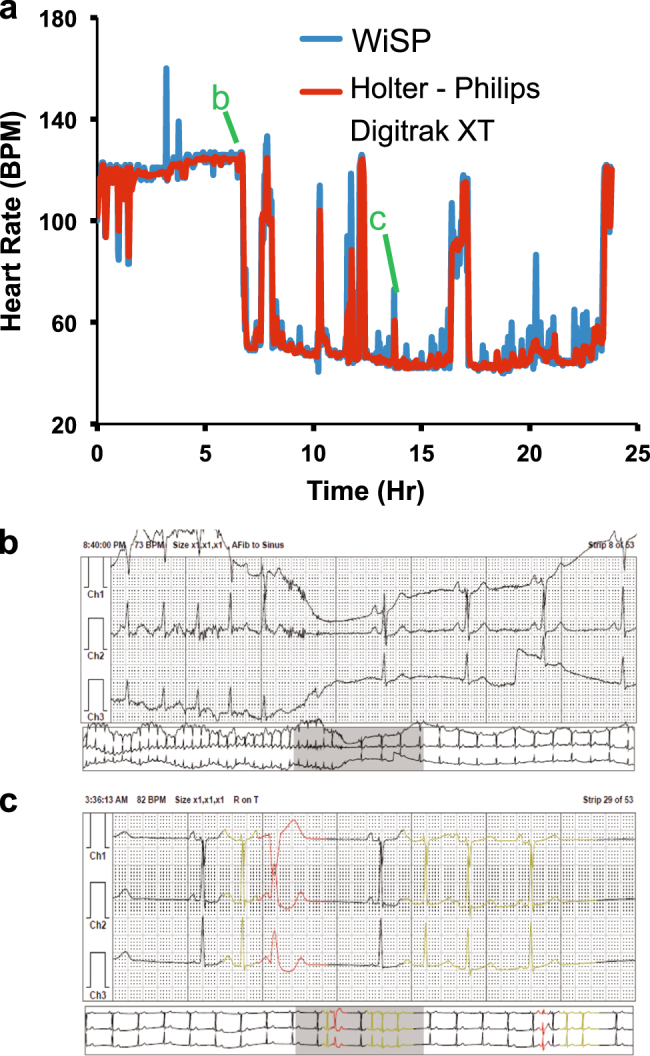
Clinical study comparing the WiSP device to standard of care Holter monitor with annotation. **a** WiSP heart rate data (2-min resolution) compared to Holter in a subject with atrial fibrillation. Annotations are detailed in sub-figures **b** and **c**. **b** Holter ECG snapshot of atrial fibrillation corresponding to the steep heart rate drop from 120 to 50 BPM. **c** Holter ECG snapshot highlighting a series of “atrial runs” corresponding to elevated heart rate

Figure 4a shows average heart rate data for a single subject captured by both the WiSP and Holter over a 24-h time period outside of the clinic. Although the captured heart rate exhibited significant dips and spikes corresponding to AF episodes, the WiSP matched the sharp changes detected by the Holter with minimal presence of motion artifacts. A few notable events were gleaned from the Holter report, as shown in Fig. 4a. We plotted the raw Holter ECG waveforms at these specific snapshots in time (Fig. 4b, c) to confirm that the rapid changes were physiological. Transient AF episodes (Fig. 4b) and short atrial runs (Fig. 4c) were identified in the ECG waveforms and found to manifest as rapid changes in average heart rate, which were accurately detected by both the WiSP and Holter.

**Fig. 5:**
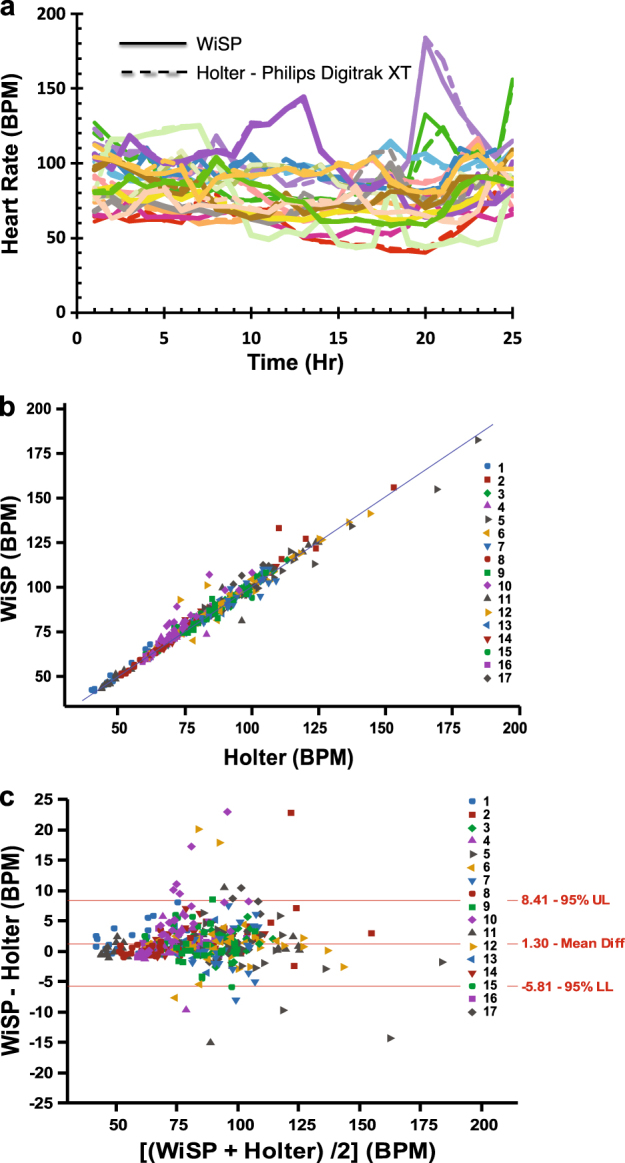
Remote monitoring study comparing the WiSP device to standard of care Holter monitor in atrial fibrillation patients. **a** Comparative analysis of WiSP (solid colored lines) vs. Holter (dashed colored lines) showing measured hourly heart rate as a function of time for multiple AF subjects (*n* = 17 patients). **b** Scatter plot comparing WiSP vs. Holter hourly heart rate data. **c** Bland–Altman plot of WiSP and Holter, comparing the hourly heart rate difference vs. hourly averaged heart rate. Comparison shows that 95% of the differences fall between +8.41 (upper limit: UL) and –5.81 BPM (lower limit: LL)

Figure 5a shows pooled WiSP and Holter heart rate vs. time data across *n* = 17 AF patients. We compared the hourly average heart rates tabulated from the Holter report to values reported by WiSP. Overall, heart rates for WiSP and Holter were strongly correlated over a broad range of heart rate zones (Fig. 5b). The WiSP device shows a slight bias of +1.3 BPM compared to the Holter. The Bland–Altman plot (Fig. 5c) shows that 95% of the differences between heart rates reported by WiSP and Holter fall between +8.5 and –5.81 BPM.

### Human factors and usability advantages

**Fig. 6:**
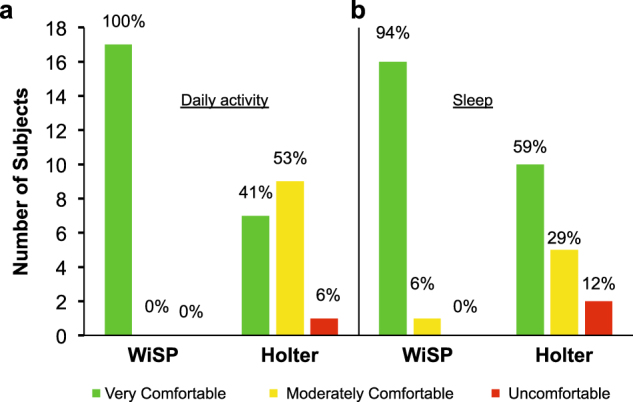
Patient exit survey results from AF clinical study participants. **a** Each patient was asked in a written survey about the “Ease of use of the WiSP device and Holter monitor” and given three choices (“very comfortable”, “moderately comfortable”, and “uncomfortable”). **b** Each patient was then asked in a written survey about their “Comfort level during sleep” and given three choices (“very comfortable”, “moderately comfortable”, and “uncomfortable”)

For subjects living with cardiovascular diseases, compliance with Holter and event monitoring devices tends to be poor even over short durations (24–48 h) of prescribed monitoring. Furthermore, studies have shown that a higher number of cardiac events may be captured wearing less obtrusive, skin-mounted biosensor patches for long durations.^39^ Patient exit surveys conducted by the clinical staff at MGH reported significantly higher patient comfort scores for the WiSP device compared to the Holter monitor during daily activity (Fig. 6a). These AF patients also reported significantly higher sleep comfort scores for the WiSP device over the Holter (Fig. 6b), suggesting that the WiSP could enhance compliance and patient experience due in large part to its imperceptible mechanical properties, small surface area, and ultrathin form factor.

## Discussion

The unique form factor, ultralow mass, flexible mechanics, and high signal fidelity of WiSP represent a major advance over existing wearable sensors. The multi-layered design of WiSP (Fig. 1a), in particular, was chosen to be compatible with standard hybrid electronics and roll-to-roll manufacturing processes to achieve disposability as a desired goal, with potential for high volume scale-up at low cost. Compared to existing packaged electronics technologies for wearable devices, the WiSP hardware is unique in its highly efficient antenna design and coordinated power management for energy harvesting and compatibility with most smartphones (Fig. 1c), elastic multi-layers of encapsulation (e.g., polyurethane) (Fig. 1d, e) and low mass, highly flexible mechanical interface with the skin (Fig. 2).

In field studies, the WiSP device compares favorably to the Polar H7 (Fig. 3), which was found to be more accurate than other competing photoplethysmogram-based wrist-worn monitors.^17^ In ambulatory care settings for AF patients, the WiSP results further show heart rate signals that match the measurements of the Holter device, with superior comfort scores (as shown in Figs. 5, 6). Taken together, these studies highlight the WiSP’s functional capabilities and enhanced wearability for remote cardiac monitoring outside of clinical environments.

Because the WiSP device is designed for daily wear and has onboard NFC capability, there are important security and wireless connectivity advantages over existing monitoring technologies. The miniaturized antenna design is compatible with many commercially available NFC-enabled smartphone models (Supplementary Table S1), whereby users can upload their encrypted cardiac data wirelessly to a cloud server, without having to return the WiSP devices to the hospital. Logged heart rate data and instantaneous ECG waveforms captured onboard the WiSP in the home and/or ambulatory environments are thus readily transmitted to care providers from anywhere in the world, without requiring wired connections to the wearable patch or in-person follow-up visits to the clinic, providing an economic benefit to both users and health-care providers.

The configurability of WiSP signal processing filters uniquely supports both episodic ECG and heart rate logging. There are many clinical applications where low-cost early screening of heart rate and episodic ECG waveforms could be utilized to significantly improve patient outcomes.^40^ For example, monitoring of heart rate signals and episodic ECG waveforms using a disposable device platform could make early or community screening services more reachable for those with limited geographical or economic access to care.^41^ Moreover, the energy-harvesting feature of the WiSP device allows multiple uses, even after the battery is fully depleted. The NFC chip onboard the WiSP device harvests additional wireless power from NFC-enabled smartphones once the smartphone is brought in close proximity to the WiSP device. As a result, the WiSP device can provide some degree of utility (episodic ECG and episodic heart rate measurements) in remote locations irrespective of battery usage.

While the small size, configurability, and conformal mechanics of WiSP make it comfortable and attractive as remote cardiac monitoring solution, the standards of care in cardiac monitoring involve more than just heart rate and ambulatory ECG measurements. Holter monitors provide 24-h recordings of ECG waveforms that skilled technicians could interpret, albeit after a few days, to quantify normal and abnormal beats. Furthermore, loop recorder devices perform beat-to-beat analysis in real time, record when abnormal rhythms are detected, and allow users to initiate a recording. Although the WiSP architecture supports a limited amount of data storage compared to Holters and loop recorders, it does allow a patient to initiate ECG recordings during abnormal rhythm events. ECG waveforms with high signal fidelity are transmitted and saved to a smartphone in real time, without expending WiSP’s limited memory supply. This allows symptomatic users that experience abnormal heart rhythm to self-initiate a recording. Future architecture enhancements of the memory storage size and expansion of battery capacity will support significantly larger data streams for more continuous mode of operation.

The WiSP device presented in this study introduces a highly miniaturized and low-power biosensing platform, which can be readily applied for remote cardiac monitoring of both endurance athletes and heart disease patients.^17,42,43^ By exploiting advances in flexible electronics and soft packaging technologies, we have established a new class of ultrathin, disposable and highly flexible wearable devices. The feasibility study results show that the WiSP device can capture heart rate (comparable to the ambulatory standard of care) and episodic ECG (comparable to commercially available patient monitors). The electromechanical design and energy harvesting functionality onboard WiSP support future opportunities for onboard beat classification (for automatic cardiac-event detection) and additional sensing, such as motion, temperature, bioimpedance and PPG for non-invasive blood pressure, thus enabling comprehensive care outside of the clinic.^42–47^ By preserving wireless connectivity to commercially available NFC-enabled smartphones, we anticipate ubiquitous deployment of WiSP as an early screening tool for ambulatory and at-home environments, where standard monitoring devices are not reachable or convenient.

## Methods

### Device construction

The WiSP prototype devices (Fig. 1) used for the current studies were assembled in-house. Populated flexible PCBs were developed in panel form and die cut to form single WiSP devices. Batteries were soldered onto the printed circuit board (PCB). Polymeric encapsulation materials with graphical layers were applied to the single units. Skin safe bioadhesive and conductive hydrogel were die cut and attached to the user-facing side of the device. Conversion was performed using commonly available die cutting tools, compatible with roll-to-roll manufacturing methodologies that allow for scalability to volume manufacturing. The WiSP devices applied in the clinical studies were packaged in low moisture vapor transmission rate bags until use.

### Internal validation study procedures

Hazard and risk analyses were performed in preparation for the clinical studies. Seven healthy volunteers had one WiSP device adhered to their skin in lead II position, and a Polar H7 strapped around their chests, near the 7th or 8th intercostal. Each subject carried a phone paired to the Polar H7, running the Polar Beat application to log heart rate data. The study coordinator used an NFC-enabled smartphone running the WiSP software application to communicate with the WiSP device and collected data at the beginning and end of the Polar wear time. Once the WiSP captured the subject's ECG morphology and began displaying heart rate, the instantaneous rate calculated by WiSP was compared to the data reported by the Polar Beat app. Heart rates were compared for up to 20 s, and noted on subject datasheets. Subjects were then allowed to conduct their normal daily activities. Each subject returned to the study coordinator for check-ins and removal of the Polar H7 approximately 4 h later. In addition to the instantaneous heart rates calculated by both devices, logged heart rates throughout the wear time were analyzed and compared after study completion. Internal validation was performed by MC10 and informed consent was obtained from each volunteer prior to the MGH study.

### Clinical feasibility study procedures

We conducted a clinical study at the Massachusetts General Hospital Cardiac Arrhythmia Unit using the WiSP devices to measure heart rate simultaneously for 24 h with a Philips DigiTrak XT Holter monitor. AF patients who were scheduled for a Holter monitor were asked if they would like to participate in this study (2015P002439 approved by the Massachusetts General Hospital Institutional Review Board). All methods were performed in accordance with the relevant guidelines and regulations of the MGH. This study ran from 30 December 2015 to 25 October 2016. Informed consent was obtained from each of the patients prior to the study. In total, 21 patients with existing clinical diagnoses of AF enrolled in this study and 17 patients completed the study. Four enrollees withdrew after signing the consent because the device did not function properly (e.g., provide good signal quality) immediately after skin-prep. These patients chose not to wait for re-application of the WiSP device by the Holter lab technician. All of the enrolled patients had existing clinical diagnoses of AF and were not compensated for their participation.

After obtaining consent from AF patients, clinical study staff prepared the patient’s skin on the torso region for WiSP device placement by abrading the skin location and cleaning with protective wipe. The WiSP device was applied to the patient in either ECG lead I or lead II configuration. The study staff used an NFC-enabled smartphone to activate the WiSP and assess the ECG signal confirming correct device placement. This initialization step powered on the WiSP and simultaneously initiated WiSP heart rate recording. With the WiSP correctly applied and assessed for high signal fidelity by the study staff, clinical technicians then applied the Holter monitor to the patient and initiated device recording.

To facilitate data stream alignment for the WiSP and Holter outputs, a timestamped photograph was taken by study staff of the recording Holter monitor device clock using the WiSP smartphone camera. As a final assessment step, the study staff held the NFC-enabled smartphone adjacent to the WiSP device to display cardiac data and visually confirm functional heart rate logging.

Patients departed the clinic and returned after 24 h in compliance with their scheduled appointment to remove the Holter monitor. At this time, the study staff held the NFC-enabled smartphone adjacent to the WiSP device to initiate wireless data transfer from the WiSP device to the phone. Study staff carefully removed the WiSP device from the patient and stored it in the supplied packaging. Patients were asked to complete an exit survey before leaving the clinic. Smartphone data was then pushed to a cloud server for analysis. Raw data files were also transferred directly from the smartphone for further analysis.

### Data analysis

Holter heart rate data was aligned with WiSP device heart rate data by matching the timestamp in the photograph taken in clinic. Because the Holter display only shows time to the minute, there may be up to ±30 s of time offset in the data. To correct for this, the WiSP heart rate data times are shifted en masse within a ±30 s window of the photograph timestamp. The WiSP device reports heart rate at 2-min intervals, whereas the Holter reports at a higher rate. To perform statistical computation, the Holter’s heart rate values at the closest time points to the 2-min cadence were selected and compared (as shown for an example subject in Fig. 4a).

The Holter continually records ECG data and lists hourly heart rate values in summary reports. The 2-min heart rate data measured by WiSP was averaged and compared to the Holter hourly data. Because multiple measurements are made per subject and there are *n* = 17 subjects in total, the data comprises a mixture of between and within-individual information. The data collected in this study is of an instantaneous value of a changing quantity, i.e., heart rate changes over 24 h. The 95.4% confidence intervals for the Bland–Altman Limits of Agreement were found by computing a one-way ANOVA and calculating the estimated variance of multiple between-method differences for the same subject and differences between subjects.^48^ The variance is computed as $\sigma _{\rm d}^2 = \sigma _{{\rm dI}}^2 + \sigma _{{\rm dw}}^2$, where $\sigma _{{\rm dw}}^2$ is the within-subject variance, mean-square residual and $\sigma _{{\rm dI}}^2$ is the variance interaction term, computed from the one-way ANOVA.^49^

This variance term, $\sigma _{{\rm dI}}^2$, is defined by the following expression:

${\sigma _{dI}^{2}} = \frac{ {(MS_{subject} - MS_{residual})}} {{\frac{{\left( { {\Sigma }m_i} \right)^2 - { {\Sigma } {m}_{i}^{2}}}}{{\left( {n - 1} \right){ {\Sigma }m_{i}}}}}}$, where *m* is the number of observations on subject *i*. The standard deviation is defined as the square root of $\sigma _{\rm d}^2$ and used to yield the 95.4% confidence intervals in Figs. 3d and 5d.

### Data availability

The data that support the findings of this study are available on request from the corresponding author [M.M.]. The data are not publicly available due to participant privacy/consent.

## Electronic supplementary material

Supplementary Figures

## Electronic supplementary material

**Supplementary information** accompanies the paper on the *npj Digital Medicine* website (10.1038/s41746-017-0009-x).
