# Supplementary material for: Highly flexible, wearable, and disposable cardiac biosensors for remote and ambulatory monitoring
Source: NPJ Digit Med. 2018 Jan 25;1:2. doi: 10.1038/s41746-017-0009-x (PMC6550217; doi:10.1038/s41746-017-0009-x)
Supplement: Supplementary file 1 — Supplementary Figures [file 41746_2017_9_MOESM1_ESM.docx]

Supplementary Information

Highly Flexible, Wearable and Disposable Cardiac Biosensors for Remote and Ambulatory Monitoring

Stephen P. Lee^1^†, Grace Ha^2^†, Don E. Wright^1^, Yinji Ma^3,4^, Ellora Sen-Gupta^1^, Natalie R. Haubrich^2^, Paul C. Branche^1^, Weihua Li^1^, Gilbert L. Huppert^1^, Matthew Johnson^2^, Hakan B. Mutlu^1^, Kan Li^4^, Nirav Sheth^1^, John A. Wright, Jr.^1^, Yonggang Huang^4^, Moussa Mansour^2^*, John A. Rogers^5^*, Roozbeh Ghaffari^1^*

**Supplementary Figures**

**Normal stresses on skin**

**Shear stresses on skin**


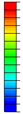


20kPa

-20kPa

Stress

1 mm

PI thickness

2 mm

0.5 mm


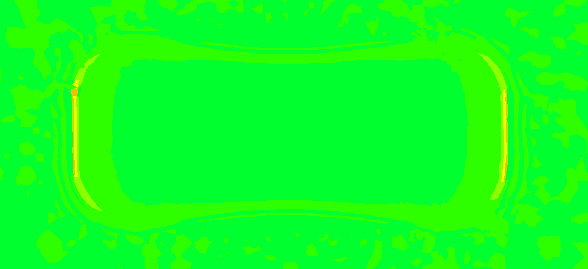

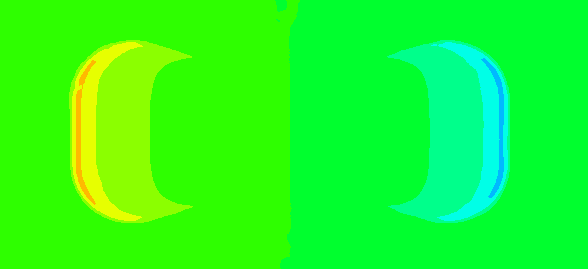


0.2 mm


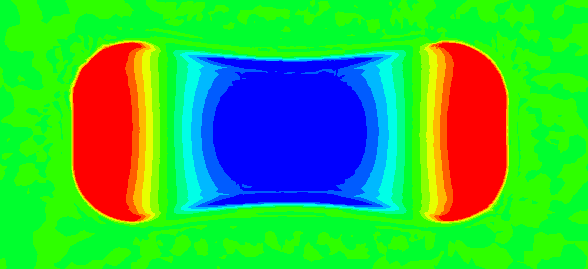

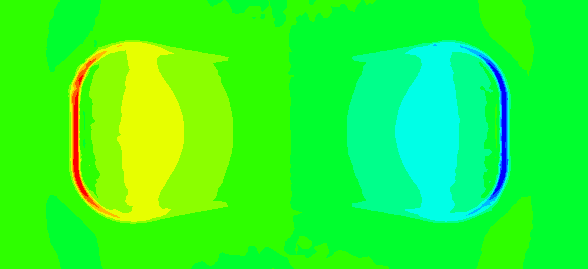

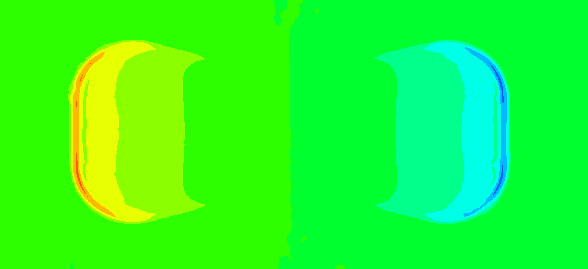

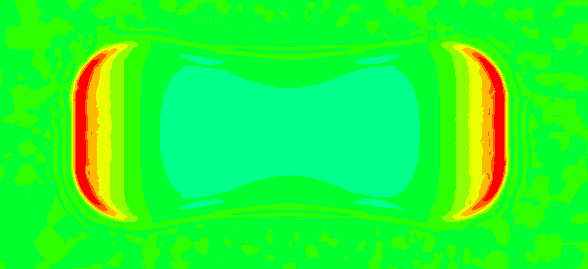

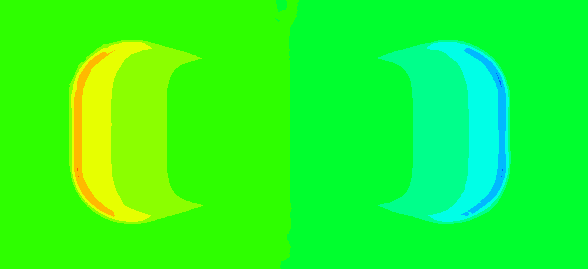

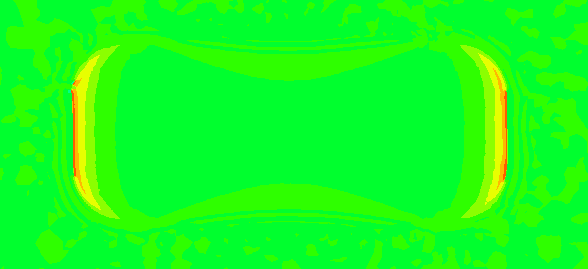


**Supplementary Figure S1 |** Computational studies detailing the effects of device thickness on bending mechanics. We examined 4 different PI thickness cases (0.2 mm, 0.5 mm, 1 mm and 2 mm) under the same applied curvature (κ=α/*L*_o_). FE results show that interfacial stresses increase with greater PI thicknesses for shear (left column) and normal (right column) stresses. The WiSP design (0.2 mm PI thickness case) exhibited the lowest shear and normal stresses in these FE simulations.

0.005 mm^-1^

**Applied curvature *κ***

0.01 mm^-1^

0.002 mm^-1^


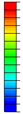


0.2%

0%

Strain

**Maximum principal strain on top surface of PI layer**


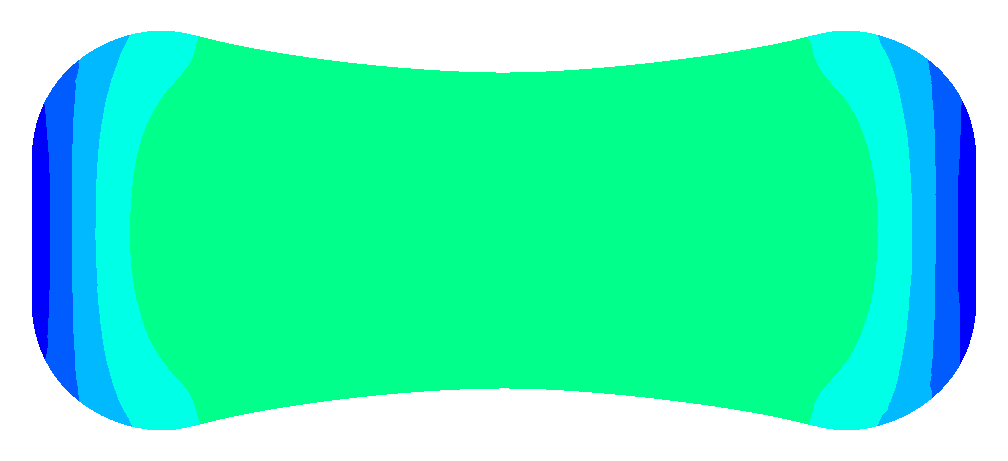

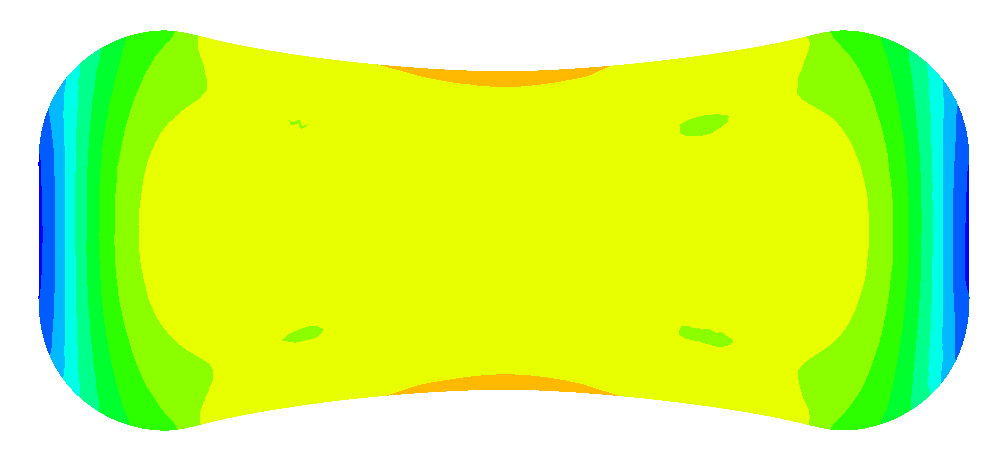

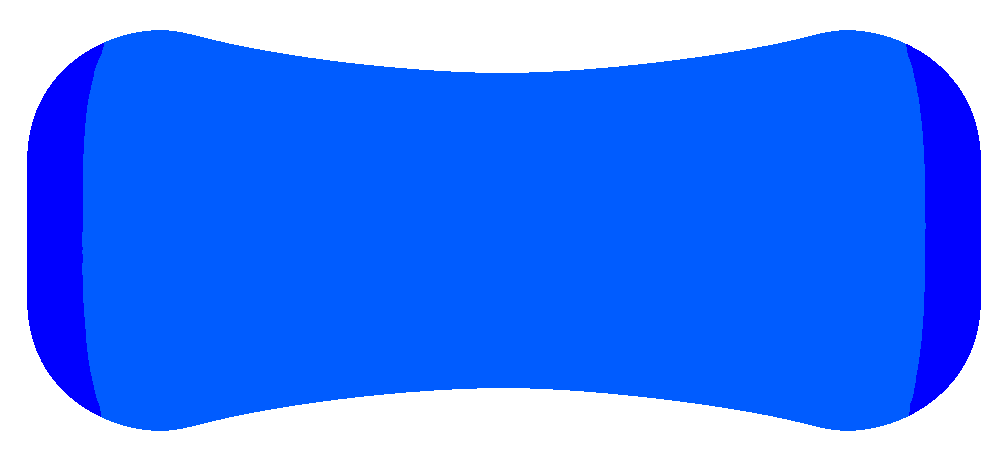


**Supplementary Figure S2 |** Computational analyses of maximum strain levels on the top of PI layer (0.2 mm thick) at the location of the flexible electronics in response to different curvature cases. For the three different applied curvatures (top: 0.002 mm^-1^, middle: 0.005 mm^-1^, bottom: 0.01 mm^-1^), the maximum principal strains are smaller than 0.2%.

| **Device Manufacturer** | **Device Model** | **Android OS** | **Kernel Version** | **NFC Availability** | **NFC Activation and Run W/O Battery** | **NFC Activation and Run W/ battery** | **NFC Streaming** | **Note** |
| --- | --- | --- | --- | --- | --- | --- | --- | --- |
| SAMSUNG | Galaxy S6 Edge | 6.0.1 | 3.10.61 | ✓ | ✓ | ✓ | ✓ |  |
| SAMSUNG | Galaxy J7 | 6.0.1 | 3.10.61 | ✓ | ✓ | ✓ | ✓ |  |
| SAMSUNG | Galaxy S5 | 6.0.1 | 3.10.9 | ✓ | ✓ | ✓ | ✓ |  |
| Google/LG | Nexus 5 | 5.1.1 | 3.4.0 | ✓ | ✓ | ✓ | ✓ |  |
| Google/LG | Nexus 5X | 7.1.1 | 3.10.73 | ✓ | ✓ | ✓ | ✓ |  |
| Google/HUAWEI | Nexus 6P | 7.1.1 | 3.10.73 | ✓ | ✓ | ✓ | ✓ |  |
| HTC | HTC ONE M9 | 6.0 | 3.10.84 | ✓ | ✓ | ✓ | ✓ | Steaming not very smooth, very good range though. |
| SAMSUNG | SPG_L300 | 4.1.2 | 3.0.31 | ✓ | ✓ | ✓ | ✓ | Streaming not very smooth, very good range though. |
| Google/HTC | Nexus 9 | 7.1.1 | 3.10.103 | ✓ | ✓ | ✓ | ✓ |  |
| Google/ASUS | Nexus 7 (2013) | 5.1.1 | 3.4.0 | ✓ | ✓ | ✓ | ✓ |  |
| Google/ASUS | Nexus 7 (2012) | 4.4.4 | 3.1.10 | ✓ | ✓ | ✓ | ✓ | Streaming not very smooth, poor range |
| SAMSUNG | NOTE II(GT-N7100) | 4.4.2 | 3.0.31 | ✓ | 🗶 | 🗶 | 🗶 | WiSP NFC standard not supported. |
| SONY | XPERIA(C1904) | 4.3 | 3.4.0 | ✓ | 🗶 | 🗶 | 🗶 | WiSP NFC standard not supported. |
| Motorola | MOTO X | 4.4.4 | 3.4.42 | ✓ | 🗶 | 🗶 | 🗶 | WiSP NFC standard not supported. |
| Google/SAMSUNG | Nexus S | 4.1.2 | 3.0.31 | ✓ | 🗶 | 🗶 | 🗶 | Can power the patch on, no communication though. |

**Supplementary Table S1 |** WiSP device and smartphone NFC compatibility matrix. NFC activation and streaming functionality tests were conducted using multiple commercially available smartphones and different versions of the Android operating systems.
